# Medial Metaphyseal Beak Angle as a Predictor for Langenskiöld Stage II of Blount's Disease

**DOI:** 10.1111/os.12760

**Published:** 2020-09-30

**Authors:** Jidapa Wongcharoenwatana, Kamolporn Kaewpornsawan, Chatupon Chotigavanichaya, Perajit Eamsobhana, Tinh Laoharojanaphand, Piyanuch Musikachart, Thanase Ariyawatkul

**Affiliations:** ^1^ Department of Orthopaedic Surgery, Faculty of Medicine Siriraj Hospital, Mahidol University Bangkok Thailand; ^2^ Department of Biochemistry, Faculty of Medicine Siriraj Hospital, Mahidol University Bangkok Thailand

**Keywords:** Blount disease, Bow leg, Langenskiöld, Predictor, Tibia vara

## Abstract

**Objective:**

To determine the medial metaphyseal beak (MMB) cut‐off angle predicting Langenskiöld stage II of Blount's disease and to study the intra‐observer and inter‐observer reliabilities of angle measurements and the influence of the experience level of observers.

**Methods:**

A retrospective study was conducted on children aged 2–4 years from January 2000 to December 2017. Children were identified through a computer‐based search. Children with Langenskiöld stage II of Blount's disease who had been initially evaluated at our institution were categorized into Blount group and children who were diagnosed with physiologic bowing were categorized into control group. Data on the patients' ages, genders, and affected sides were collected. The MMB angles were measured on standing anteroposterior radiographs of the knees. The angle was formed between one line drawn parallel to the medial cortex of the proximal tibia, and a second line running from the intersection of the first line with the proximal tibial metaphysis through to the most distal point of the MMB. Measurements were independently performed by six observers. All observers repeated the measurements 2 weeks after they were first done.

**Results:**

There were 148 legs from 79 children (48 males and 31 females) with an average age of 28.6 months. The average MMB angle of the Blount group was 128.52° ± 5.38° (*P*‐value <0.001) and of the control group was 114.45° ± 4.89°. The average femorotibial angle of the Blount group was 15.48° ± 6.81° (*P*‐value <0.001) and of the control group was 7.71° ± 7.94°. The receiver operating characteristic curve showed that an MMB angle >122° (sensitivity 92.7%; specificity 97.0%) was associated with Langenskiöld stage II. The intraclass correlation coefficient of the intra‐observer reliability ranged from 0.93–0.97, and the inter‐observer reliability was 0.93.

**Conclusions:**

By using anteroposterior (AP) radiographs of the knee, the MMB angle is a potential radiographic parameter to distinguish between Langenskiöld stage II of Blount's disease and physiologic bowed legs, with an MMB angle >122° predicting Langenskiöld stage II.

## Abstract 2

The medial metaphyseal beak (MMB) angle is a potential radiographic parameter, with high intra‐and inter‐observer reliabilities, to distinguish between Langenskiöld stage II of Blount's disease and physiologic bowed legs, with an MMB angle > 122° predicting Langenskiöld stage II.

## Introduction

Bowing deformity of the lower extremities is commonly seen in children in the first years of life. Parents concerned about their child's excessive bowing condition frequently visit orthopaedists for evaluation. Differential diagnosis of bowed legs in children between age 1 to 3 years are physiologic and pathologic conditions. Physiologic bowed legs are a consequence of normal growth and development in both the coronal and transverse planes^1^, ^2^, ^3^. Children present with obvious bowed legs around 2 years old, followed by knock‐kneed at the age 2 to 3 years. Then, develop slightly valgus knee similar to normal alignment of the lower extremity in adults by 6 to 7 years of age^1^, ^4^. Pathologic bowing of the lower extremity may be due to a variety of causes, including: persistent physiologic varus; renal osteodystrophy, dietary vitamin D deficiency, and vitamin D‐resistant rickets; focal fibrocartilaginous defects of the proximal tibial metaphysis; and skeletal dysplasia. However, the most common cause of pathologic bowing in children is infantile tibia vara or Blount's disease^5^, ^6^, ^7^. These differential diagnoses are easily distinguished from true infantile Blount's disease by history of symptoms, short stature, skeletal dysplasia features, and obvious radiographic findings.

Blount's disease is an acquired asymmetrical growth disorder of the proximal medial tibial physis and epiphysis which results in a progressive genu varum deformity of the lower extremities. The condition was first described in detail by Blount^5^ in 1937. He purposed the term “osteochondrosis deformans tibiae” and identify two forms of this disorder which were infantile and adolescent type. However, this condition was often referred to as Blount's disease. Prevalence of Blount's disease in young children with significant bowed legs in the United States is less than 1%.^8^

Infantile Blount's disease is typically seen in children between the ages of 2 to 5 years and is characterized by varus deformity of the proximal tibia in an otherwise healthy child. Bilateral involvement is found in approximately 50% of cases^5^ and boys are affected more often than girls^5^, ^6^. The etiology of infantile Blount's disease remains unknown. Deceleration of growth occurs at the posteromedial area of proximal tibial physis, which results in varus, flexion, and internal rotational deformity. Histological study of the affected physis has shown disruption of normal columnar architecture of the physis, fibrous tissue replace physeal cartilage, and osseous bridging between the epiphysis and metaphysis in severe cases^5, 9^.

Definitive diagnosis of Blount's disease is based on progressive clinical bowed legs with the presence of characteristic radiographic changes of proximal and medial tibial physis as described by Langenskïold^6^. In 1964, Langenskïold and Riska^6^ purposed a radiographic classification based on a study of Swedish patients and described the six progressive roentgenographic stages of the infantile type of Blount's disease and these were widely used as factors determining disease prognosis. They stated that spontaneous correction often occurs in patients with stage II lesions and occasionally in those with stage IV lesions.

Langenskiöld's classification is based on a cascade of different degrees of epiphyseal depression and metaphyseal fragmentation of the proximal medial tibial epiphysis^6^, ^9^, ^10^, ^11^. Langenskiöld's classification has been confirmed by many roentgenographic studies done on Blount's disease between 1952 and 1963^6^, ^9^, ^10^, ^11^. Despite the variability in both inter‐ and intra‐observer measurements, Langenskiöld's classification system is still very commonly used by orthopaedic surgeons^6^, ^12^, ^13^.

The characteristics of Langenskiöld stage II of Blount's disease have been described as a sharp depression of the physeal line medially and a medial metaphyseal beak (MMB). However, the degree of the MMB required for categorization as Langenskiöld stage II of Blount's disease is subjective and is dependent on each assessor. Since the radiographic finding of a normal proximal medial tibial metaphysis in young children with physiologic bowing can also present with a mild beak, it is sometimes difficult to distinguish Langenskiöld stage II of Blount's disease. Based on only one radiograph at the initial visit in children aged between 1 and 3 years, distinguishing between physiologic bowing and Blount's disease was very difficult. This leads to delayed or missed diagnoses and treatment. The early and accurate distinction between these two conditions is of great importance because, while physiologic bowing does not require any treatment, children with Langenskiöld stage II of Blount's disease should be treated with a knee‐ankle‐foot orthosis (KAFO) as soon as possible in order to avoid permanent medial physeal arrest and restore normal growth^9^, ^11^, ^14^.

To our knowledge, there is no previous study describing how much of the beak is needed for a diagnosis of Langenskiöld stage II of Blount's disease. MMB angle can be a potential radiographic parameter to distinguish between Langenskiöld stage II of Blount's disease and physiologic bowed legs in younger children. Therefore, the aims of the current research were to: (i) establish radiographic parameters and determine the cut‐off angle of the MMB predicting Langenskiöld stage II of Blount's disease; (ii) study the intra‐observer and inter‐observer reliabilities of MMB measurements; and (iii) study the influence of the experience level of observers on the intra‐observer reliability.

## Materials and Methods

This study was approved by the hospital's institutional review board. A retrospective study was conducted on data from children aged 2–4 years between January 2000 and December 2017. Demographic data (comprising patients' ages, sex, BMIs, affected sides and Langenskiöld stages) were collected. Inclusion criteria were as follows: (i) children aged 2–4 years; (ii) who visited our outpatient department with bowed leg problem; and (iii) had standing anteroposterior (AP) radiograph of the knees. Exclusion criteria were as follows: (i) radiographic evidence of a metabolic bone disease or focal fibrocartilaginous dysplasia; (ii) other orthopaedic or medical problems that were related to lower extremities; (iii) had received previous treatment; or (iv) had incomplete medical records and/or radiographic data.

We divided the children into a Blount group (Langenskiöld stage II) and a control group (physiologic bowed legs). In the case of the Blount group, the children were identified through a computer‐based search for children with Langenskiöld stage II of Blount's disease who had been initially evaluated at our institution. Children with physiologic bowing in the control group were identified through a computer search for all children seen in the outpatient department of our institution who had been initially evaluated between 2 and 4 years of age.

Radiographic measurements were made from the initial radiographs for each child in each group. The measurements of the femorotibial angle and the MMB angle were evaluated from a standing AP radiograph of the knees. A good quality AP view was determined by the position of the tibial spine, which needed to be at the same position as the intercondylar notch of the femur. The fibular head had to be 0.5 to 1.5 cm below the tibial plateau, with a quarter of the fibula head overlapping the tibia.

Both femorotibial angle and MMB angle were independently measured by six observers (two senior pediatric orthopaedic surgeons, a pediatric orthopaedic surgeon, a pediatric orthopaedic fellow, an orthopaedic chief resident, and a junior orthopaedic resident) using radiograph archiving and communication system (PACS) software. To decrease the incidence of recall bias, the observers repeated the measurements on all of the radiographs 2 weeks after they were initially done.

### *Medial Metaphyseal Beak Angle*

**Fig. 1:**
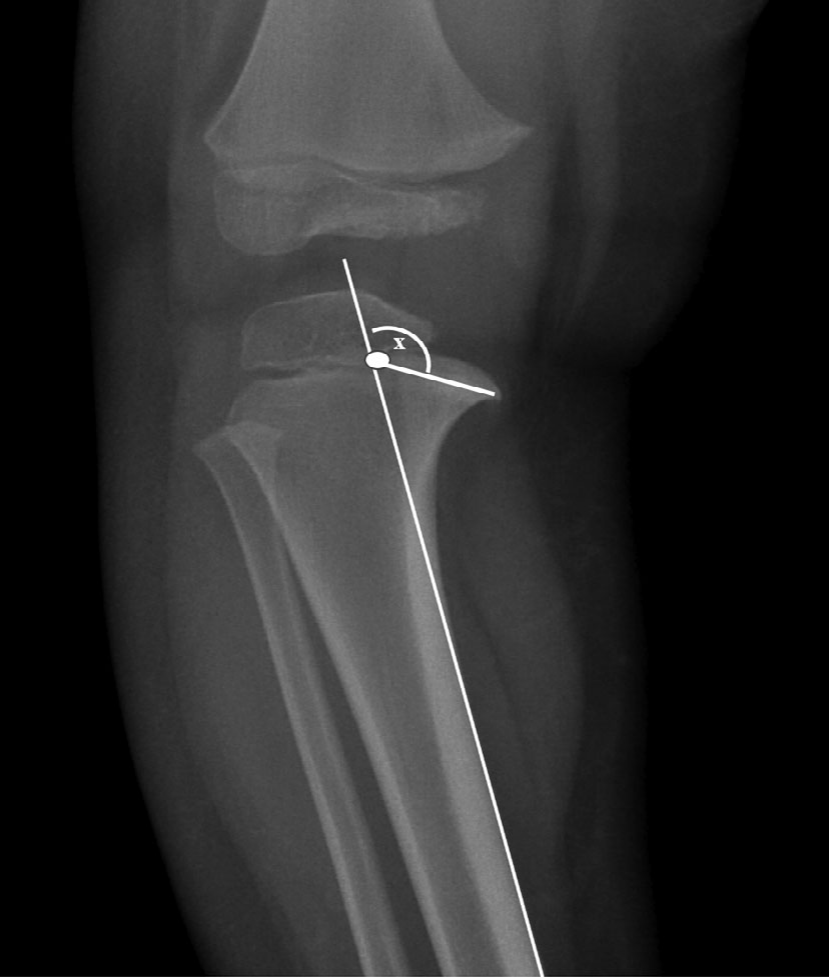
The MMB angle (x) was formed between two lines. **Line 1** was drawn parallel to the medial cortex of the proximal tibia, whereas **line 2** was drawn from the intersection point of the first line with the proximal tibial metaphysis through to the most distal point of the medial metaphyseal beak.

Medial metaphyseal beak was a medial metaphysis which depressed and had a beak‐like protuberance of bone medially. The medial metaphyseal beak angles were determined using the radiograph archiving and communication system software, and they were measured on the AP radiographs of the knees. The angle was formed between one line drawn parallel to the medial cortex of the proximal tibia, and a second line running from the intersection of the first line with the proximal tibial metaphysis through to the most distal point of the MMB (Fig. 1). The MMB angle was used to determine the severity of metaphyseal beak and to distinguish between Langenskiöld stage II of Blount's disease and physiologic bowed legs. However, there was no previous literature that categorized the severity of MMB angles in order to differentiate between these two conditions.

### *Femorotibial Angle*

Femorotibial angle was defined as the angle between the anatomical axis of the femur and the tibia in the AP radiographs of the knee joint. The knee is defined varus when this angle is ≥0° with the apex oriented laterally, valgus if this angle ≥0° with the apex oriented medially.

### *Data Analysis*

To determine the cut‐off angle predicting Langenskiöld stage II, a statistical analysis was performed using the receiver operating characteristic (ROC) curve and MedCalc software, version 17.6 (MedCalc Software bvba, Ostend, Belgium). The cut‐off angle was selected from the values that had the highest sensitivities and specificities. Comparisons between groups were performed using the two‐tailed t‐test for continuous variables with a normal distribution, and the Mann–Whitney U test for continuous variables with an abnormal distribution.

The intra‐observer and inter‐observer reliabilities of the MMB angles were analyzed using intraclass correlation coefficients based on a 95% confidence interval for absolute agreement. A correlation coefficient of 0–0.20 indicated poor reliability, 0.21–0.40 signified fair reliability, 0.41–0.60 represented moderate reliability, 0.61–0.80 denoted good reliability, and ≥0.81 demonstrated excellent reliability. Fisher's exact test, the independent t‐test, and the Mann–Whitney U test were used to compare the differences between age groups, and between genders and sides. Means and standard deviations were used to describe the variability of the data. *P*‐values of <0.05 were defined as being statistically significant. The software program used to analyze the data was PASW Statistics for Windows, version 18.0 (SPSS Inc., Chicago, IL, USA).

## Results

### *Demographic Study*

There were a total of 148 legs from 79 children (48 males, and 31 females). Children were divided into two groups: a “Blount” group (Langenskiöld stage II) and a “control” group (physiologic bowed legs). The 82 legs in the Blount group had an average age of 27.93 ± 5.02, while the average age of the 66 legs in the control group was 29.24 ± 4.32 (*P*‐value = 0.114) **(**Table 1 **)**. The average femorotibial angle of the Blount group was 15.48 ± 6.81° and of the control group was 7.71° ± 7.94° (mean difference 7.77 (95% CI [5.36 to 10.19]); *P*‐value <0.001; Table 2 **)**.

**TABLE 1:** Patients' characteristics

	Blount (n = 82)	Control (n = 66)	*P*‐value
Gender			
Male	47 (57.32%)	43 (65.15%)	0.398
Female	35 (42.68%)	23 (34.85%)	
Age (months)	27.93 ± 5.02	29.24 ± 4.32	0.114
BMI (kg/m^2^)	24.82 ± 4.32	24.77 ± 4.93	0.956
Side			
Left	46 (56.10%)	27 (40.91%)	0.072
Right	36 (43.90%)	39 (59.09%)	

*P‐value < 0.05 was considered statistically significant.

**TABLE 2:** Medial metaphyseal beak and femorotibial angles of the two groups

	Blount (n = 82) (Mean ± SD [Minimum–Maximum])	Control (n = 66) (Mean ± SD [Minimum–Maximum])	Mean difference (95%CI)	*P*‐value
Medial metaphyseal beak angle (°)	128.52 ± 5.38 (111.90–140.10)	114.45 ± 4.89 (104.20–123.40)	14.06 (12.37 to 15.75)	<0.001*
Femorotibial angle (°)	15.48 ± 6.81 (1–39)	7.71 ± 7.94 (2–34)	7.77 (5.36 to 10.19	<0.001*

* *P*‐value < 0.05.

### *Medial metaphyseal beak angle*

**Fig. 2:**
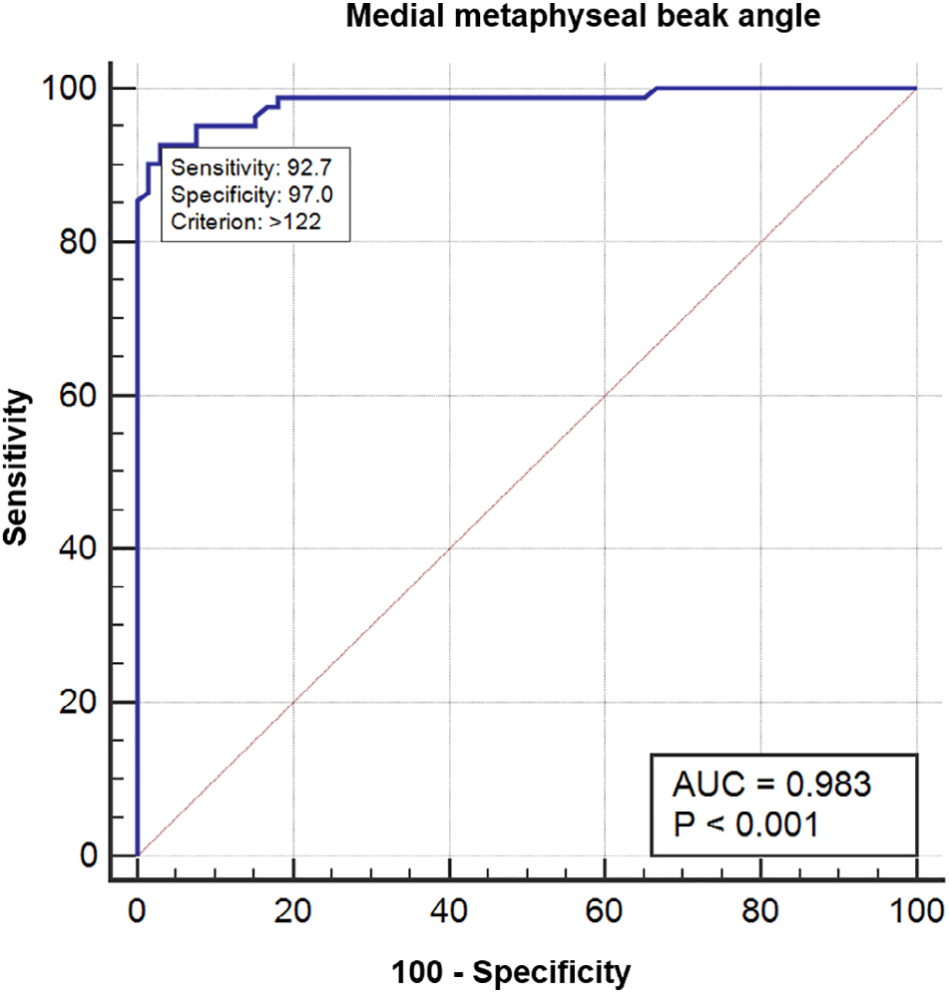
Receiver operating characteristic (ROC) curve for the medial metaphyseal beak angles.

The average MMB angle of the Blount group was 128.52° ± 5.38°, while that of the control group was 114.45° ± 4.89° (mean difference 14.06 [95% CI 12.37 to 15.75]; *P*‐value <0.001; Table 2 **)**. The ROC curve showed that an MMB angle >122° (sensitivity 92.7%; specificity 97.0%) was associated with Langenskiöld stage II **(**Fig. 2 **)**. The intraclass correlation coefficient of the intra‐observer reliability ranged from 0.93 to 0.97, while the inter‐observer reliability was 0.93 **(**Table 3 **)**.

**TABLE 3:** The intraclass correlation coefficients with 95% confidence intervals of intra‐ and inter‐observer reliabilities of the medial metaphyseal beak angles, categorized by six observers

	ICC (95% CI)
Intra‐observer correlation	
A senior pediatric orthopaedic surgeon (1)	0.94 (0.92, 0.96)
A senior pediatric orthopaedic surgeon (2)	0.97 (0.96, 0.98)
A pediatric orthopaedic surgeon	0.96 (0.94, 0.97)
A pediatric orthopaedic fellow	0.93 (0.90, 0.95)
An orthopaedic chief resident	0.96 (0.95, 0.98)
A junior orthopaedic resident	0.94 (0.92, 0.96)
Inter‐observer correlation	0.93 (0.92, 0.95)

CI, confidence interval; ICC, intraclass correlation coefficient.

### *Demographics of Medial Metaphyseal Beak Angle*

Mean ± SD of MMB angle compared between Blount and control groups classified by gender, age, BMI, and affected side were shown in Table 4. The results showed statistically significant differences in MMB angles between Blount and control groups when categorized into gender, age, BMI, and affected side in both Blount and control groups.

**TABLE 4:** Mean value of the medial metaphyseal beak angle categorized by gender, age group, BMI and affected side

	MMB in Blount (°) (Mean ± SD [Minimum–Maximum])	MMB in Control (°) (Mean ± SD [Minimum–Maximum])	Mean difference (95%CI)	*P*‐value
Gender				
Male	126.63 ± 4.96 (111.90–140.10)	114.68 ± 4.67 (104.70–123.40)	11.95 (9.93 to 13.97)	<0.001*
Female	131.07 ± 4.91 (120.00–138.10)	114.05 ± 5.36 (104.20–122.20)	17.02 (14.28 to 19.75)	<0.001*
Age (months)				
<24	130.75 ± 5.09 (123.40–138.10)	114.80 ± 7.85 (104.20–120.80)	15.95 (9.21 to 22.68)	<0.001*
≥24	128.03 ± 5.36 (111.90–140.10)	114.45 ± 4.74 (104.70–123.40)	13.59 (11.82 to 15.36)	<0.001*
BMI (kg/m^2^)				
<23	129.16 ± 6.05 (120.00–140.10)	114.26 ± 6.06 (104.20–123.40)	14.89 (10.80 to 18.98)	<0.001*
≥23	128.21 ± 5.13 (111.90–137.70)	115.36 ± 4.38 (107.80–121.90)	12.85 (10.01 to 15.70)	<0.001*
Side				
Left	129.05 ± 5.16 (120.20–140.10)	115.60 ± 4.79 (105.90–122.20)	13.44 (11.01 to 15.88)	<0.001*
Right	127.85 ± 5.66 (111.90–138.00)	113.67 ± 4.86 (104.20–123.40)	14.19 (11.76 to16.61)	<0.001*

* *P*‐value < 0.05.

MMB, Medial metaphyseal beak angle.

## Discussion

This study demonstrated that an MMB angle >122° can predict Langenskiöld stage II of Blount's disease with a high sensitivity and a high specificity. Children with physiologic bowing had lower values for their MMB angles than children with Langenskiöld stage II.

The differential diagnosis of bowed legs in children between 1 and 3 years of age include both physiological and pathological conditions. Pathological bowing of the leg may be due to a variety of causes, but the most common is infantile tibia vara, better known as Blount's disease^5^, ^7^, ^10^, ^15^.

Because of the poor treatment outcomes for Blount's disease if diagnosis and treatment are delayed, early diagnosis of the disease is highly beneficial. For one thing, it will avoid proximal medial tibial physeal arrest and restore normal growth by unloading the proximal medial tibial growth plate with bracing or osteotomy before 4 years of age^16^, ^17^, ^18^, ^19^, ^20^. Moreover, it will serve to discourage unnecessary follow‐ups and inappropriate repeat radiographic evaluations of children with physiologic bowing.

The six stages of the Langenskiöld classification system are widely used by orthopaedic surgeons to categorize Blount's disease patients. However, its use is subjective and dependent on each assessor. Moreover, variability has been shown in both the inter‐ and intra‐observer reliabilities^6^, ^12^, ^13^.

### *Medial Metaphyseal Beak Angle*

An MMB is one of the Langenskiöld stage II characteristics. However, as a proximal medial tibial metaphysis in young children with physiologic bowing can also present with a mild MMB, it is sometimes difficult to distinguish Langenskiöld stage II of Blount's disease. Therefore, in some cases, there is a high chance of disagreement among surgeons about the Langenskiöld staging. However, no previous literature has evaluated the degree of the MMB needed to predict Langenskiöld stage II of Blount's disease. Therefore, this study focused on the measurement of the MMB angle to distinguish between physiologic bowing and Langenskiöld stage II.

**Fig. 3:**
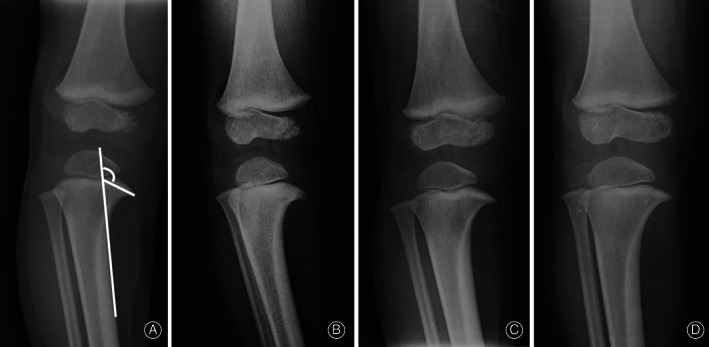
Series of radiographs from physiologic bowed legs patient at age of (A) 21 months showed MMB = 120°, (B) 24 months showed MMB = 120°, (C) 27 months showed MMB = 117°, (D) 31 months showed MMB = 112°.

**Fig. 4:**
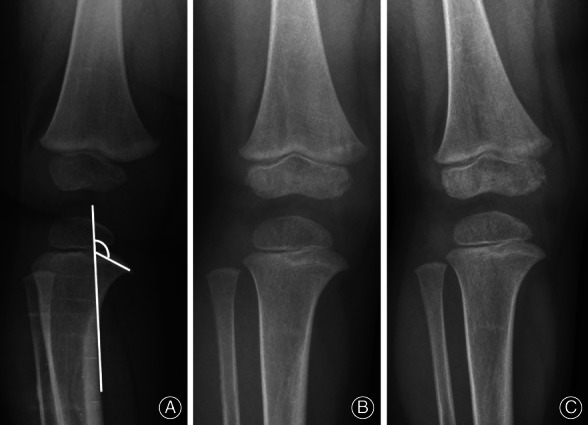
Series of radiographs from physiologic bowed legs patient at age of (A) 16 months showed MMB = 118°, (B) 28 months showed MMB = 113°, (C) 33 months showed MMB = 109°.

**Fig. 5:**
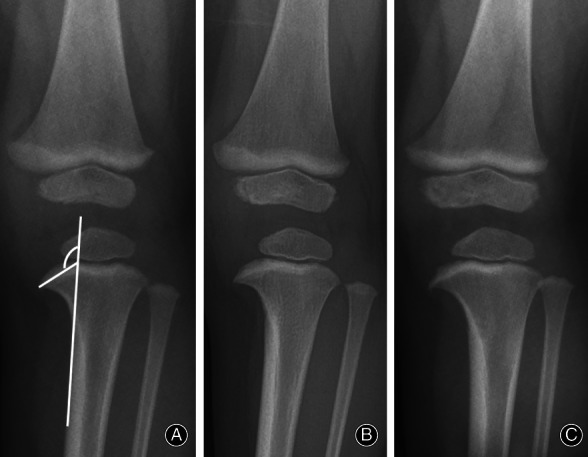
Series of radiographs from Blount's patient at age of (A) 16 months showed MMB = 126°, (B) 20 months showed MMB = 125°, (C) 24 months showed MMB = 126°.

The load on medial side of proximal tibia causes depression of medial metaphysis area. With a higher load in Blount's disease patients, it can lead to a greater depression of medial metaphysis and more severe medial metaphyseal beak compared with physiologic bowing. We found that an MMB angle >122° was associated with Langenskiöld stage II with a high sensitivity and a high specificity. This result supports the proposition that the MMB angle is a potential radiographic parameter for predicting Langenskiöld stage II of Blount's disease. Series of radiographs of Blount's disease and physiologic bowing patients with MMB angles were shown in Figs 3, 4, 5.

In our study, the children with physiologic bowing showed lower values for the MMB angle than those with Langenskiöld stage II. This correlated with the clinical finding that the femorotibial angles of the children with physiologic bowing displayed less varus deformity of the knee. This therefore produced a lower load on the proximal medial tibial metaphysis and a reduction in the MMB deformity.

### *Level of Experience*

There is no published evidence of the relationship between experience level and the reliability of the measurement of the MMB angle. The current study was carried out by six observers with different levels of experience. Both their intra‐ and inter‐observer reliabilities showed high intraclass correlation coefficients, which demonstrated that the measurements of the MMB angles did not significantly differ between the different experience levels of observers.

The limitations of this study were its small sample size (due to the low prevalence of the disorder) and its selection bias (typical of a retrospective study).

### *Conclusions*

By using AP radiographs of the knee, the MMB angle is a potential radiographic parameter to distinguish between Langenskiöld stage II of Blount's disease and physiologic bowed legs, with an MMB angle >122° predicting Langenskiöld stage II.

## Authorship declaration

All authors listed meet the authorship criteria according to the latest guidelines of the International Committee of Medical Journal Editors, and that all authors are in agreement with the manuscript.
